# Per-organ assessment of subject-induced susceptibility distortion for MR-only male pelvis treatment planning

**DOI:** 10.1186/s13014-018-1090-2

**Published:** 2018-08-15

**Authors:** Carri Glide-Hurst, Siamak Nejad-Davarani, Steffen Weiss, Weili Zheng, Indrin J. Chetty, Steffen Renisch

**Affiliations:** 1 Department of Radiation Oncology, Henry Ford Cancer Institute, Detroit, MI 48202 USA; 2 Department of Digital Imaging, Philips Research Laboratories, 22335 Hamburg, Germany; 3 Department of Radiation Oncology, William Beaumont Hospital, Royal Oak, MI 48073 USA

## Abstract

**Background:**

Patient-specific distortions, particularly near tissue/air interfaces, require assessment for magnetic resonance (MR) only radiation treatment planning (RTP). However, patients are dynamic due to changes in physiological status during imaging sessions. This work investigated changes in subject-induced susceptibility distortions to pelvic organs at different bladder states to support pelvis MR-only RTP.

**Methods:**

Pelvises of 9 healthy male volunteers were imaged at 1.0 Tesla (T), 1.5 T, and 3.0 T. Subject-induced susceptibility distortion field maps were generated using a dual-echo gradient-recalled echo (GRE) sequence with B_0_ field maps obtained from the phase difference between the two echoes acquired at several bladder volume states (3–4/subject, 32 overall). T2 turbo spin echo images were also acquired at each bladder state for organ delineation. Magnet central frequency was tracked over time. Distortion map differences and boxplots were computed to characterize changes within the clinical target volume (CTV), bladder, seminal vesicles, and prostate volumes.

**Results:**

The time between the initial and final B0 maps was 42.6 ± 13.9 (range: 13.2–62.1) minutes with minimal change in magnet central frequency (0.02 ± 0.05 mm (range: − 0.06 – 0.12 mm)). Subject-induced susceptibility distortion across all bladder states, field strengths, and subjects was relatively small (1.4–1.9% of all voxels in the prostate and seminal vesicles were distorted > 0.5 mm). In the bladder, no voxels exhibited distortions > 1 mm. An extreme case acquired at 3.0 T with a large volume of rectal air yielded 27.4–34.6% of voxels within the CTVs had susceptibility-induced distortions > 0.5 mm across all time points.

**Conclusions:**

Our work suggests that subject-induced susceptibility distortions caused by bladder/rectal conditions are generally small and subject-dependent. Local changes may be non-negligible within the CTV, thus proper management of filling status is warranted. Future work evaluating the impact of multiple models to accommodate for extreme status changes may be advantageous.

## Background

Images of high geometric fidelity enable accurate delineation of disease extent and proximity to organs at risk (OARs), which are essential for high-precision radiation treatment planning (RTP). However, the current standard of care for RTP is based on CT simulation (CT-SIM), which does not provide adequate soft tissue discrimination. This limitation has been addressed by registering diagnostic magnetic resonance imaging data (MRI) to CT-SIM datasets. This existing CT-SIM-based workflow relies on target and OAR definition on MRI and a transfer of contours to CT via image registration. MRI-CT co-registration introduces geometrical uncertainties of ~ 2 mm for prostate patients [1, 2]. Importantly, these errors are systematic, persist throughout treatment, shift high dose regions away from the target [3] and could lead to a geometric miss that compromises tumor control. Thus, there has been a strong interest to move toward an MR-only RTP workflow to eliminate redundant CT scans (reducing radiation dose, patient time, and imaging costs), streamline clinical efficiency, and importantly, MR-only RTP would entirely circumvent systematic co-registration uncertainties [4–6].

MRI has been limited by known geometric distortions arising from two major components: system-level (arising from gradient nonlinearity (GNL) in the spatial encoding gradients [7, 8] and B_0_ field inhomogeneities) and patient-level (chemical shift artifacts and susceptibility) [9]. Currently, GNL distortion corrections are built into the MRI reconstruction software. While our preliminary results at 1.0 T showed that residual GNL—after vendor corrections—was non-negligible and required additional corrections before MR-only RTP can be implemented [10], closed bore magnets have shown clinically acceptable GNL within the useable field of view (FOV) [11, 12]. Patient-level distortions, on the other hand, are object- and sequence-dependent and increase with field strength, requiring patient-specific corrections. Susceptibility differences are most apparent near tissue/air interfaces due to local variations in the induced magnetic field and have been reported to be up to 4 mm at the sinus/tissue interface in the brain at 3.0 T [9].

Few studies have investigated the impact of subject-induced susceptibility distortion in the pelvis. Recently, Tyagi et al. measured distortions using B_0_ maps (i.e., the difference of two phase images measured at different echo times (TEs)) and found that the distortions within the prostate were < 0.5 mm for a 20 patient cohort, however other organs and multiple time points were not studied [13]. Stanescu et al. and Lundman et al. performed simulation studies to investigate susceptibility distortions in the pelvis and found the magnitude was lower than other sites such as lung or head and neck [14, 15]. In another study, Kemppainen et al. investigated patient specific geometric distortions in the PTV, bladder and rectum of four pelvic cancer subjects on a 1.5 T scanner and reported a distortion of less than 1 mm in these organs [16] . However, in addition to susceptibility induced distortions, pelvic patients may experience changing transient anatomy (e.g., bladder and rectal filling) that can occur during long MR image acquisition times. These status changes may result in geometric inconsistencies as well as local subject-induced susceptibility distortion changes (termed “susceptibility distortions” in this work) at air/tissue interfaces. This work sought to characterize changes in susceptibility distortions in pelvic organs arising from transient anatomy during MRI examinations to support an MR-only RTP workflow in the pelvis.

## Methods and materials

### Subject population

**Fig. 1:**
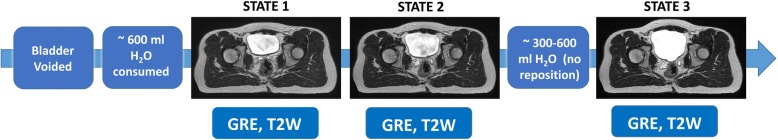
Experimental design for variable bladder filling study. Abbreviations: GRE = Gradient recalled echo sequence, T2W = T2-weighted turbo spin echo sequence. This figure shows T2W images of subject S7 (scanned on the 1.0 T scanner) with the states 1–3 corresponding to actual acquisition times of 12, 32 and 55 min, respectively, after the time the first survey was acquired. For cases where 4 bladder states were acquired, two MRI datasets were acquired after additional water was consumed by the subjects

Nine healthy male volunteers (age: 43 ± 10.1 years, range 25–61 years; weight: 78.8 ± 9.6 kg) were consented to research studies managed by local Institution Review Boards. Three volunteers were scanned using a large, rigid 8-element phased array coil on a 1.0 T Panorama High Field Open Magnetic Resonance System (1.0 T Panorama HFO; Philips Healthcare, Best, The Netherlands) equipped with flat table top (Civco, Orange City, IA) and external laser system. Three healthy male volunteers were scanned at 1.5 T (Philips Achieva, 32-element torso coil) and the last three on a 3.0 T (Philips Ingenia, 32-element coil in the patient bed and anterior array) with standard concave table tops and using the magnet bore lasers for positioning. Figure 1 illustrates the experimental design for image acquisition for all cases. In brief, subjects were instructed to void their bladder and then consumed ~ 600 ml of water. They were positioned supine and sequences were acquired at several states: (State 1) empty bladders, (States 2–3) partially full bladders, and (States 3–4) full bladders at each field strength. The total imaging time on all scanners was ~ 45 min. Considering that lower field strengths have longer acquisition times, only three T2-weighted and B0 image sets were acquired at 1.0 T. However, for 1.5 T and 3.0 T, shorter sequence times allowed for the acquisition of additional bladder states within this time. Therefore, 3–4 evaluable states were used per subject based on field strength with a total of 32 evaluable time points for the cohort. After two full sets of acquisitions were acquired, an additional 300–600 ml of water was consumed while the subject was on the table with no repositioning as illustrated by Fig. 1. In this manner, a range of bladder filling states (i.e. between interim and extreme states of bladder filling) could be investigated. A similar workflow can also be used to create a library of radiation treatment plans for actual patients, similar to a study that was previously done for cervical cancer patients [17].

### Subject-induced susceptibility distortion maps

For each time point, T2-weighted (T2) turbo spin echo images were also acquired as these are most commonly used for delineation of the prostate gland [18, 19] and cervical cancer [20]. The bandwidth of the T2 sequence was chosen to optimize signal-to-noise ratio (SNR) as is common for this task. Field mapping was performed using a dual-acquisition gradient echo (GRE) sequence. FOVs were chosen to cover the entire body laterally and the pelvic region of interest in feet-head direction. Shim settings were held constant for each subject across the entire acquisition time with sequence parameters shown in Table 1. At each field strength, GRE TEs were set to yield two in-phase images, higher bandwidths were used to minimize the chemical shift related distortion, and voxel sizes were selected based on tradeoffs between resolution and acquisition time, which were also consistent with the literature [9]. Maps of the phase difference (*∆ϕ*) were reconstructed by evaluating the phase after complex division of the complex data from two echoes. The phase of the complex ratio equals to the phase difference between the two images. Phase difference maps were unwrapped using Prelude in the Functional Magnetic Resonance Imaging of the Brain (FMRIB) Software Library (FSL, Analysis Group, FMRIB, Oxford, UK). B_0_ field maps were obtained from the phase difference *∆ϕ* evolved between the two echoes with time difference □₤TE: $\Delta  {B}_0=\frac{\Delta  \phi }{2\uppi \upgamma \Delta  TE}$(γ is the gyromagnetic ratio of hydrogen) [21]. This study focuses on a Cartesian non-echo-planar T2 sequence for delineation. For such a sequence, susceptibility-based local changes of the resonance frequency directly transfer into local displacements along the frequency encoding or read-out direction, whereas the phase encoding direction remains unaffected. To convert the B_0_ field map to a distortion map (x-displacement) based on the T2 sequence, the following equation was used:

1 $$ \Delta  \mathrm{x}=\frac{\Delta  {\mathrm{B}}_0}{{\mathrm{G}}_x}=\frac{\Delta  {f}_0}{BW_f}\Delta  {\mathrm{V}}_{\mathrm{x}}=\frac{\Delta  \upphi}{2\uppi \Delta  TE\ast {BW}_f}\Delta  {\mathrm{V}}_{\mathrm{x}} $$

with following T2 sequence parameters: G_*x*_ is the readout gradient, ∆x is x-displacement along the frequency-encoding direction; *BW*_f_ is the acquisition pixel bandwidth (Hertz(Hz)/pixel) in the frequency encoding direction and *∆*V_x_ is pixel size in the frequency encoding direction. Other sequences will require sequence-specific corrections that are beyond the scope of this work and detailed in the literature [21].

**Table 1:** MRI sequence parameters used in this study. The dual-acquisition gradient echo (GRE) sequence was used for field mapping and the T2 images were acquired for delineation of the organs. x, y and z indicate the axes in the left-right, anterior-posterior and superior-inferior directions respectively

Field Strength (T)	Sequence	Acquisition ΔV_x_/ΔV_y_/ΔV_z_ (mm)	Bandwidth (Hz/pixel)	α	TR (ms)	TE1(ms)	TE2(ms)	FOV (mm)	Turbo Factor
1.0	GRE	1.41/1.41/3	975	10°	15.87	6.91	13.81	474.19 × 474.19 × 300	–
	T2	1.49/1.6/2.5	202	90°	5070	80	–	397.89 × 397.89 × 250	24
1.5	GRE	2.9/2.9/4	543	10°	11.19	4.61	9.22	416.90 × 416.90 × 200	–
	T2	0.96/0.96/2.5	207	90	4620	100	–	180 × 180 × 84	17
3.0	GRE	2.9/2.9/4	1078	10°	2.30	2.30	4.61	416.90 × 416.90 × 200	–
	T2	0.96/0.96/2.5	218	90°	4295	100	–	180 × 180 × 84	17

It is important to note that the subjects were not repositioned between acquisitions. To further minimize effects of any displacement of the subjects between acquisitions, magnitude images from the first echo of all GRE and T2 datasets were registered to the first image set using FMRIB’s Linear Image Registration Tool module in FSL [22, 23]. Six parameter (translation and rotation) rigid registration was performed using nearest neighbor interpolation and mutual information as the cost function. Visual inspection of bony alignment was performed for each case. The resultant transformations were then applied to the corresponding B_0_ field maps to perform voxel-by-voxel comparisons.

To quantify susceptibility effects in organs of interest, the prostate, seminal vesicles, and bladder were delineated on the corresponding T2-weighted dataset at of each bladder state by a single physician. The clinical target volume (CTV) was defined as the union of the prostate and proximal 1 cm of the seminal vesicles as consistent with our clinical practice [24]. The delineated organs were used as a mask on the corresponding distortion map to quantify susceptibility-induced distortions for each organ across images from all bladder states via box plots and corresponding descriptive statistics. One-way analysis of variance (ANOVA) (with posthoc Bonferroni adjustments to address multiple comparisons) were performed to evaluate statistically significant differences in susceptibility-induced distortion between physiological states (i.e., empty vs. partially full bladder, empty vs. full bladder, etc.) within each subject.

To employ the distortion measurement methodology proposed here, the resonant frequency (f0) of the scanner is assumed to be constant over the entire imaging acquisition session. However, scanner resonant frequencies may change due to temperature changes, warming of electronics, or variations in the subject composition (i.e. presence of air, tissue, etc.) in or near the volume used for f0 determination [25]. To characterize these variations, the f0 was tabulated from the image header of the B0 map sequence for each subject and field strength. The f0 is the optimal RF excitation frequency that is automatically measured at the beginning of each MRI scan based on a subvolume centered within the imaging FOV. These data are logged by the scanner in the DICOM header and were used in this work to assess the stability of the f0-determination. For the shim procedure, the shimming is held constant for each volunteer. The frequency (in Hz) was converted to units of T2 distortion using Eq. 1.

## Results

### Central frequency drift

**Fig. 2:**
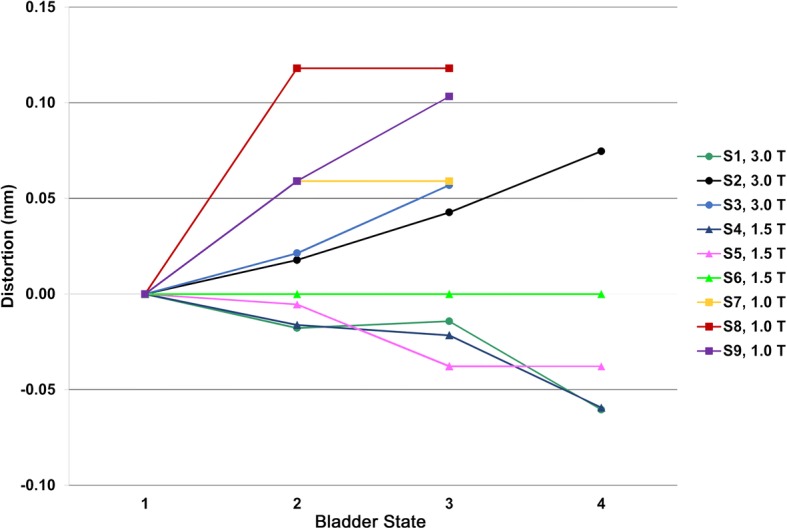
Temporal changes in magnet central frequency over time for the 9 subjects (S1-S9) (converted from Hz to mm using Eq. (1) and using T2 parameters listed in Table 1)

On average, the time between the initial and final B0 maps (time point 3 or 4 depending on the subject) was 42.6 ± 13.9 (range: 13.2–62.1) minutes. As shown in Fig. 2, the central frequency drift effect over the imaging sessions across all magnets was generally negligible (0.02 ± 0.05 mm (range: − 0.06 – 0.12 mm)). The maximum f0 shift (16 Hz) occurred for the 1.0 T scanner over ~ 50 min, which was converted to a T2 distortion of 0.12 mm for time points 2 and 3 (Subject 8 in Fig. 2). Despite the larger central frequency drift for this case, the corresponding distortion in the PTV was shown to increase at the 2nd time point and subsequently decrease at the 3rd time point.

### Magnitude and distribution of patient-specific distortions

**Fig. 3:**
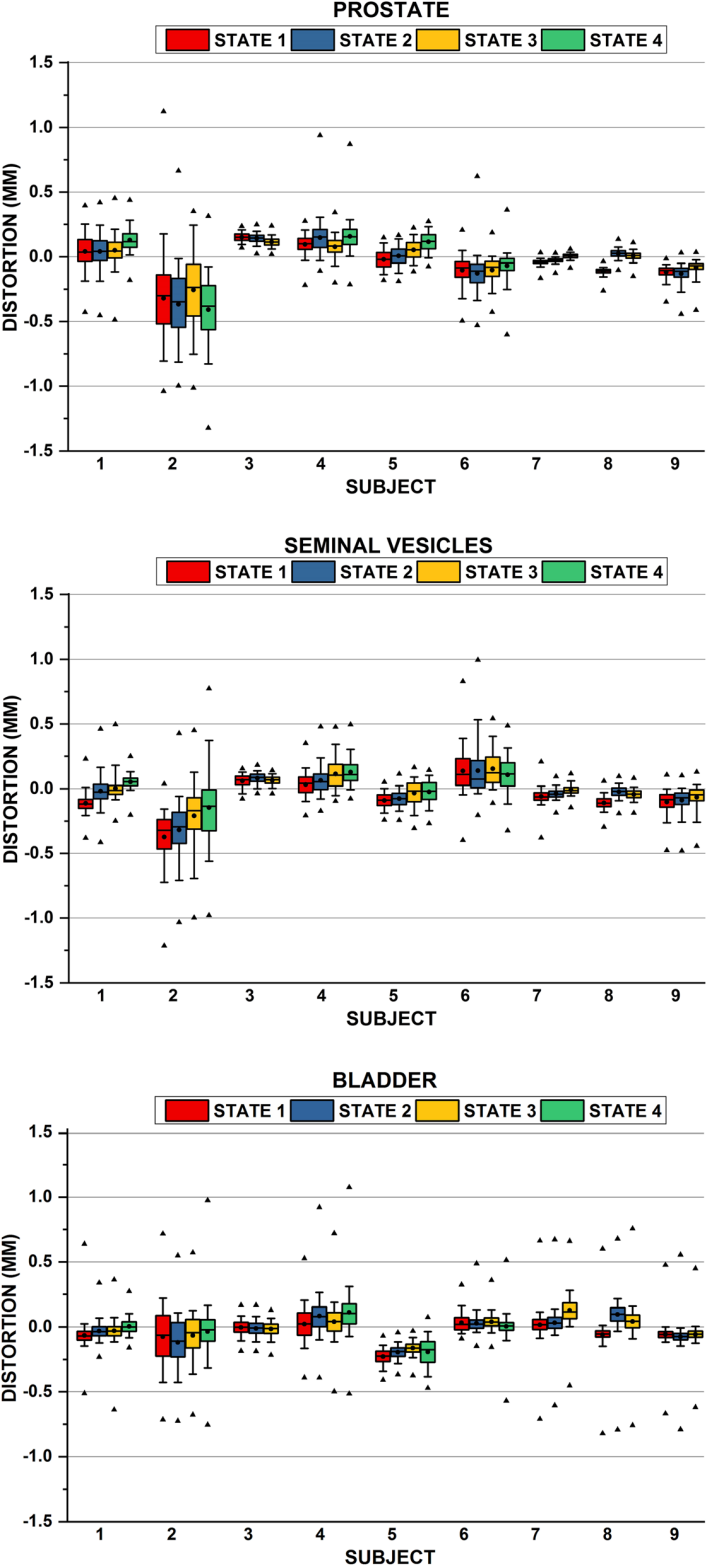
Distribution of patient-induced susceptibility effects in the prostate (top), seminal vesicles (middle), and bladder (bottom) for bladder state (S1 Empty bladder, S3 or S4 Full bladder). Boxplots, line, and dot indicate the interquartile range (25–75%), median, and mean respectively. Whiskers indicate 5th and 95th percentile, ▲ indicate the minimum and maximum values. Subjects 1–3 were acquired at 3.0 T, 4–6 were acquired at 1.5 T, and 7–9 were acquired at 1.0 T

Figure 3 summarizes the overall distortions within the prostate and seminal vesicles. Subjects 7, 8, and 9 acquired at 1.0 T demonstrated the smallest susceptibility distortions. Notably, Subject 2, acquired at 3.0 T, not only showed the largest susceptibility but also the largest variation of the magnitude of the bladder volume. There was a significant effect of bladder state on organ-specific distortion at the *p* < 0.05 level for 37 out of 42 combinations of matched states for the prostate, seminal vesicles, and bladder. All pairwise comparisons between the initial and final states were statistically different for all organs studied. Even so, the overall magnitude of distortion yielded only 1.4–1.9% of all voxels in the prostate and seminal vesicles distorting > 0.5 mm across the cohort. When Subject 2 was excluded, the overall distortion was negligible for all other cases (< 0.2% and < 0.03% of all voxels distorted > 0.5 mm within the seminal vesicle and prostate, respectively). In the bladder, no voxels exhibited distortions > 1 mm. Again, the largest distortion in the bladder occurred for Subject 2 with 1.3–2.2% of voxels distorting > 0.5 mm across all bladder states studied. For this case shown in Fig. 5, large pockets of rectal air were observed at the first state that did not fully resolve over the entire acquisition.

**Fig. 4:**
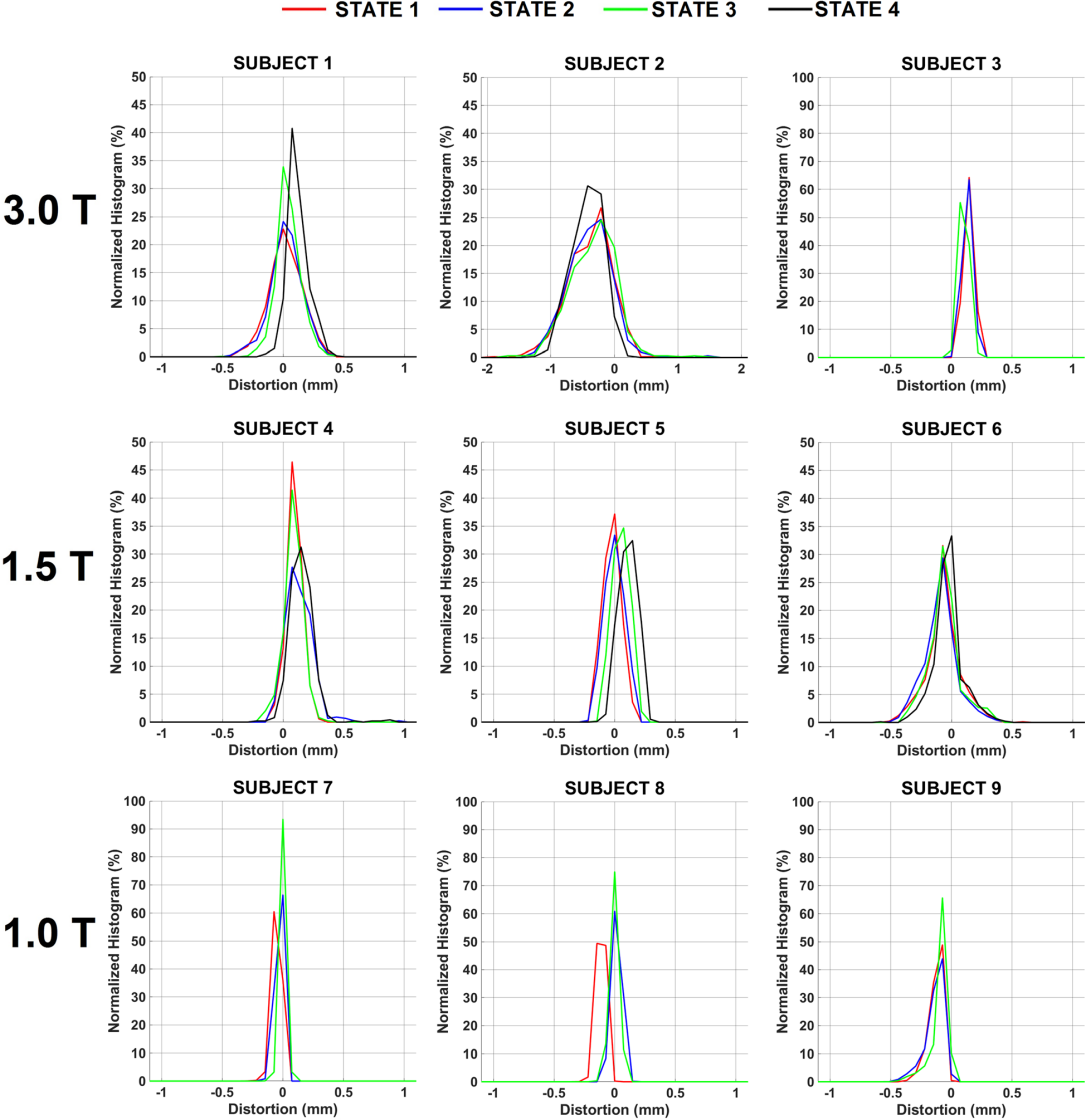
Histogram of distortions normalized to the total number of voxels within the CTV for 9 subjects to characterize local subject-specific susceptibility distortions arising due to changes in bladder/rectal filling status

**Fig. 5:**
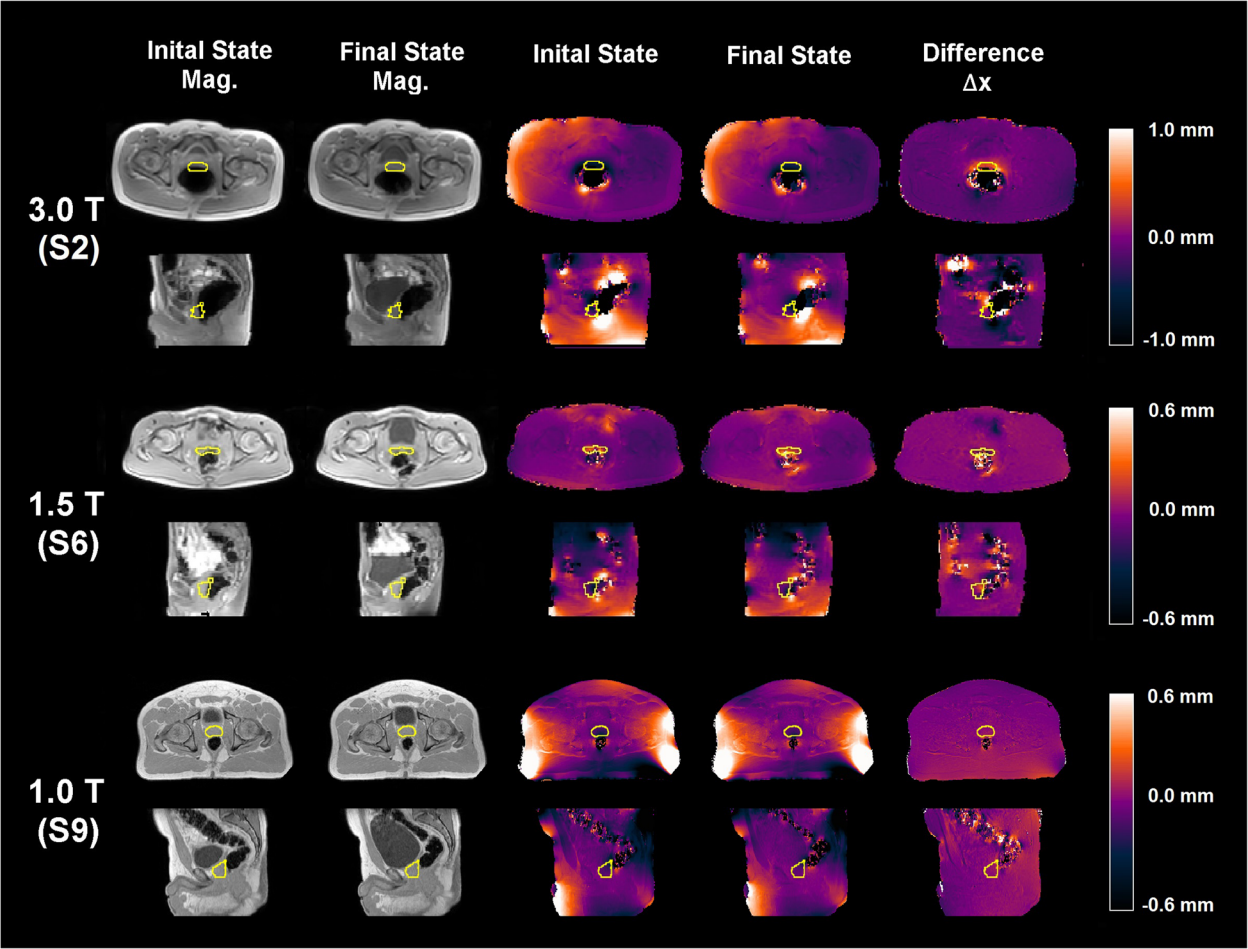
Magnitude images of the dual echo MRI data of initial (column 1) and final (column 2) bladder states for three subjects (S) used to generate subject-induced susceptibility distortion maps. Corresponding B0 maps with off-resonances converted to distortions are shown in columns 3 and 4, respectively. Clinical target volumes (CTVs, prostate + proximal 1 cm of seminal vesicles) are delineated. Distortion difference maps (difference ∆X) are shown in the final column (final minus initial) with the distortion scale shown in millimeters

Overall, distortion magnitude was small although the voxel-based distributions illustrate that the overall ranges and distortion changes based on bladder state were subject-specific. Figure 4 summarizes the CTV distributions for all time points in the cohort. Figure 5 illustrates local distortion differences arising from rectal/bowel changes induced by bladder filling for a subject at each field strength to highlight different characteristics in the subject population. For Subject 2, changes in bowel air were observed, with large amounts of rectal air influencing distortions within the CTV (~ 27.4 to 35.7% of the voxels yielded patient-specific susceptibility distortions > 0.5 mm, largely at the rectum/bowel gas and tissue interfaces). Subject 6 was selected at 1.5 T as it showed the largest distortions at this field strength (range = 0.8 to 1.1 mm within the CTV). Finally, Subject 9 data acquired at 1.0 T illustrated that a very large increase in bladder volume did not influence the rectal status for this case, and thus susceptibility-induced distortions within the CTV remained stable whereas local distortions in the bowel were more apparent.

## Discussion

As we move toward MRI-only treatment planning, it becomes important to characterize distortion magnitude that may arise in this new workflow. This work sought to quantify patient-specific distortion in the pelvis and considered geometric changes that may occur in the delineated organs based on susceptibility induced distortions due to bladder and rectal status changes occurring over long MRI acquisition times. The results of this work may also extend to MR-guided radiation therapy, particularly at higher field strengths where subject-induced distortions are expected to be higher. While MRI acquisition times were long in this study, the baseline variation of the f0 determination during that time was negligible (0.02 ± 0.05 mm, ~ 0.12 mm in the most extreme case). In our study, the resonance frequency (f0) was held constant across the acquisition session. In the pelvis, compositional changes in bowel and rectal gas may occur over an image acquisition session and thus impact the volume used for f0 determination, thus it is recommended that this should be held constant for magnetic resonance simulation examinations. Recently, Wang et al. performed repeat acquisition of field maps for 17 brain subjects and found a within-subject standard deviation of ~ 0.2 mm displacement in the frequency-encoding direction of 3D T1-weighted images [9]. The authors concluded that this variation was small and possibly due to eddy current decay in their 3.0 T magnet, which can also be a contributing effect to variations in susceptibility over time. In our study of the male pelvis, physiological status changes across 3 field strengths caused more marked impact on local susceptibility than what was observed for the repeat measurements in the brain.

While statistically significant differences were found in 37 out of 42 comparisons for the prostate and seminal vesicles, overall distortion magnitude was quite low (< 2% of all voxels distorted > 0.5 mm). Subject 2 exhibited the largest distortions in the prostate and seminal vesicles. When Subject 2 was omitted from the cohort, < 0.2% of all prostate and seminal vesicles voxels across all states had subject-induced susceptibilities > 0.5 mm. This work considered subject-induced distortions in several male pelvic organs, however the femoral heads were not analyzed. Recent work published by Adjeiwaah et al. simulated expected subject-induced distortions with similar bandwidths at 244 Hz/pixel and found that median distortions were ~ 0.5 mm with ~ 75% of all distortions in the femoral heads were < 1 mm [26]. Also, Tyagi et al. evaluated patient-induced susceptibility distortion in the pelvis in a cohort of 20 prostate patients acquired at 3.0 T. They found the mean distortion within the prostate for a single acquisition dataset to be − 0.2 mm (range: − 0.62–0.35 mm) [13]. These results were of similar magnitude to our population results although their calculations were based on full bladder conditions controlled by a Foley catheter. In their study, the mDixon sequence with a relatively high acquisition pixel bandwidth used to generate synthetic CTs for treatment planning using commercially available software was evaluated whereas our study was focused on the T2-weighted sequences used for delineation. Bandwidths used in this study were selected to optimize SNR and are consistent with literature on T2-weighted pelvis acquisitions [13, 26]. Eq. 1 highlights that distortion due to susceptibility effects is dependent on the frequency-encoding bandwidth and field strength. This effect can be reduced by increasing the acquisition bandwidth at the expense of reducing SNR. Our results can be extrapolated to other T2-weighted sequences with different acquisition parameters based on Eq. 1.

Susceptibility distortions scale with field strength and size of the inhomogeneity [14]. This was observed in our study, where the magnitudes of local distortions in the CTV were largest for subjects at higher field strengths and with larger rectal gas volumes. Thus, it can be expected that when the volume of rectal air increases, susceptibility-related distortions in the abutting CTV would also increase, which was consistent with our results. It was found that subjects with no rectal air near had minimal susceptibility distortion changes over different bladder volume states. Similarly, subjects acquired at the lowest field strength of 1.0 T had no appreciable differences in susceptibility effects using the different models. However, in a worst-case scenario (Subject 2 acquired at 3.0 T with large rectal status changes between time points), distortion differences within the CTV were appreciable. This suggests that had the first set of susceptibility maps been used to correct for susceptibility distortions, corrections would have been applied to anatomy that was no longer at the same state and location.

One limitation of this study was that data was obtained on healthy volunteers. However, intermediate and extreme physiological states, ranging from empty to full bladders, were considered. Fig. 5 illustrated that the presence of large pockets of rectal air introduced the largest susceptibility distortions although almost all voxels distorted < 1 mm. Recently, Adjeiwaah et al. simulated dosimetric differences due to patient-induced susceptibility at various bandwidths for 17 prostate cases and found clinically acceptable plan quality with relative dose differences of < 0.5% in the PTV [26], suggesting that distortions may not yield dosimetric differences.

This work was not designed to address GNL distortions, although applying vendor distortion corrections and centering the area of interest near magnet isocenter can reduce GNL to < 1 mm for most magnets [11, 27]. While distortion correction maps can be applied, our work has illustrated the potential distortion changes arising from different organ states, thereby complicating the use of a single distortion correction map in the pelvis. This suggests that if corrections are to be made, an important quality assurance step will include evaluating spatial alignment of air-filled organs. Because patient-induced susceptibility distortions will arise near tissue/air interfaces such as the prostate/rectal interface where high dose gradients typically exist, it may be advantageous to correct for the distortions via an inverse warping and Jacobian scaling technique as described in the literature [10, 28]. This interface is critical in RTP because margins are typically reduced to decrease the risk of rectal toxicity [29] and volumetric image-guided radiation therapy alignment is emphasized in this region [24]. Another approach would be to ensure proper margins are applied to accommodate this uncertainty. Overall, organ-specific subject-induced distortions quantified in this work were small but may be clinically significant based on the location.

## Conclusions

Our work suggests that subject-specific distortion differences caused by transient gas are generally small and subject-dependent. However, local changes may be non-negligible near interfaces, thus proper management of filling status is warranted.

## Abbreviations

ANOVA: Analysis of variance
B_0_ map: Map of the B_0_ field created using the difference of two phase images measured at different echo times
CT-SIM: Computed tomography simulation
CTV: Clinical target volume
f0: Resonant frequency
FMRIB: Functional magnetic resonance imaging of the brain
FOV: Field of view
FSL: FMRIB Software library
GNL: Gradient nonlinearity
GRE: Gradient echo
Hz: Hertz
kg: Kilograms
mm: Millimeter
MR: Magnetic resonance
OAR: Organ at risk
PTV: Planning target volume
RTP: Radiation treatment planning
SNR: Signal-to-noise ratio
T: Tesla
T2: T2-weighted
TE: Echo time
